# The economics of home support services in Ireland: exploring complex issues of healthcare sustainability and aging populations

**DOI:** 10.3389/fpubh.2025.1602617

**Published:** 2025-06-02

**Authors:** Elizabeth Morrow, Mary Lynch

**Affiliations:** Faculty of Nursing and Midwifery, Royal College of Surgeons Ireland, Dublin, Ireland

**Keywords:** home care, home support, workforce development, workforce economics, aging population

## Abstract

**Background:**

Home support is a critical yet under-recognized component of public health and aging policy, with economic perspectives often overlooked in workforce and system planning. This paper explores the economics of home support services in Ireland, against a backdrop of increasing demand for home care driven by aging populations and workforce supply challenges.

**Aims:**

To provide a comprehensive overview of the economic challenges faced by the home support sector in Ireland, particularly in relation to workforce sustainability, population health, and quality service delivery. To explore international best practices and case studies that can further inform the development of home support models.

**Methods:**

Utilizing both qualitative and quantitative population, labor force, and health service data, the analysis of the Irish context investigates three critical areas: (1) the rising demand and funding of home support, (2) cost comparisons with institutional care, and (3) the economic implications of workforce expansion. Systematic literature review of the international evidence used a structured search of electronic databases (Web of Science, MEDLINE, CINHAL) using key terms (“home support,” “workforce development,” and “economic or cost”) to identify a range of recent (published 2015–2025) and relevant case studies to inform policy development.

**Results:**

Findings indicate that Ireland’s market-driven approach, heavily reliant on approved private providers, exacerbates issues like low wages, job insecurity, and high staff turnover, which negatively impact service quality. Implications for healthcare workforce policy include improving wages and working conditions, establishing career pathways and professional development, and increased government investment. Recommendations for policy include making strategic investments in workforce stability and better integration of home support with informal care systems to enhance service delivery.

**Conclusion:**

Policymakers can inform themselves about the economic considerations for developing a robust home support system in the context of aging populations. Further research is needed into the assumptions and validity of financial decisions to ensure services are sustainable.

## Introduction

Home support plays a critical role in population and public health, enabling people to age with dignity and independence at home. Economic perspectives on home support have not been fully integrated into policy and workforce planning, or population and public health strategies. In Ireland and other high-income countries, the home care sector is shaped by the work of informal carers, labor market dynamics, government funding, private providers, wage structures, and workforce stability. With Ireland’s population aged 65 and over projected to rise from 14.5% in 2019 to nearly 22% by 2040 (1), and a shift away from long-term residential care, demand for statutory home support is growing (2, 3). However, low wages, job insecurity, and limited career progression threaten the sector’s long-term growth and contribution to healthcare system sustainability (4).

In this paper, the term “home support service” refers to formal paid assistance, while acknowledging that 60–70% of home care in Ireland is provided by unpaid family or informal carers, alongside an unknown number of privately paid arrangements (5, 6). A “home support worker” (HSW) is trained to assist clients in their homes. However, HSWs are unregulated (6), leading to varied job titles (e.g., home help, personal care attendant) and responsibilities (7). While traditionally focused on personal care and domestic support, home support services (or adult social care) internationally are evolving to include delegated medical tasks, raising concerns about client safety and the optimal models of care provision in aging populations (8, 9).

Economic research highlights the challenges of meeting the costs or growing demand for community-based care, with studies emphasizing government financial pressures and workforce challenges. For example, Colombo et al. (10) identify sustainability concerns in OECD care systems, stressing the need for workforce support to maintain service quality. While Brennan et al. (11) examine market-driven care models in Nordic and liberal welfare states, showing how privatization often leads to low wages and job insecurity- challenges also evident in Ireland’s mixed public-private home support model (Statutory Home Support Scheme). Despite these concerns, limited research has explored the economics of home support or the impact on healthcare systems (7).

Economically, home support is often more cost-effective than institutional care, reducing long-term healthcare expenditure and enabling individuals to remain at home in their communities for longer (2). Increased investment in home support could alleviate pressures on hospitals and residential care facilities while improving overall service efficiency, however there is a dearth of robust economic research on the complex issues surrounding the economics of home support, such as the opportunity costs of service expansion and upskilling HSWs (5, 9).

To address the challenges in Ireland, the authors have collaborated with sector leaders (see Acknowledgements) to advocate for a structured HSW career pathway (12) and funding for empirical economic studies to inform workforce development (13). Policy engagement has included the production of a white paper and roundtable policy discussion in 2024 (14). The present paper builds on these initiatives, focusing on economic perspectives. This paper does not aim to address the full complexity of the issues surrounding home support in Ireland. Instead, it focuses on three interconnected economic issues: (1) the rising demand and funding of home support, (2) cost comparisons with institutional care, and (3) the economic implications of workforce expansion. An economic perspective helps to highlight the interconnected nature of these issues, ensuring that solutions are considered within the broader context of Ireland’s evolving care system (15).

## Aim

The aim of this paper is to provide a comprehensive analysis of the economic challenges facing the home support sector in Ireland, with a focus on workforce sustainability, population health, and the delivery of high-quality services. Additionally, it aims to review relevant research literature, examining international best practices and case studies from other countries seeking to expand and enhance their home support services. The research aims to inform the development and enhancement of home support schemes in Ireland and elsewhere.

### Research questions

This paper addresses four research questions:

1. How is the increasing demand for home support services in Ireland influencing current funding models, and what are the implications for future resource allocation?
2. How do the costs and outcomes of home support services compare to institutional care in Ireland, and what are the economic trade-offs involved?
3. What are the economic implications of expanding the home support workforce in Ireland, particularly regarding wages, retention, and service sustainability?
4. What international models of home support demonstrate effective economic and workforce strategies, and how can these inform Ireland’s policy development?

## Methods

### Approach

The approach is to provide an overview of Ireland’s home based care model and economic considerations, rather than a statistical analysis of financial data. The approach aligns with established methodologies for economic scoping and policy-relevant system analysis (16, 17), where the focus is on identifying key assumptions and evidence gaps rather than conducting detailed statistical modelling. Economic estimations are used to highlight assumptions and areas where further empirical research is needed to support policy development. This approach ensures that the article provides valuable insights for policymakers and decision making about aging populations, while highlighting the gaps in the current evidence base that require further investigation (17). Using a range of qualitative and quantitative, statistical reports, policy and economic data, the analysis includes examining supply–demand dynamics, home support service costs, and cost-benefits of home support. Data sources include sector statistics, literature, government reports, and the research literature. The main contribution of the paper is to provide economic insights and policy recommendations to strengthen the Irish home support sector, with broader implications for other countries facing similar challenges.

To investigate the international evidence, a systematic literature review and case study approach (18), was adopted to identify relevant empirical and evaluative studies. Sources were selected using a structured and reproduceable literature review process developed by Petticrew and Roberts (19), to ensure methodological rigor in identifying and synthesizing relevant evidence. Sources from the research literature were selected based on three primary criteria: (a) relevance to home support services and workforce planning; (b) recency, with a focus on literature published within the past 10 years; and (c) geographical applicability, prioritizing studies from Ireland and countries with comparable aging populations. Relevant studies were identified using structured searches of electronic databases Web of Science, MEDLINE, CINHAL using key terms such as “home support,” “workforce development,” and “economic or cost,” limited to articles published Jan 2015- Jan 2025.

## Results

The findings are presented in four sections to address each of the research questions.

### Rising demand and funding of home support in Ireland

#### Policy to expand home support

The introduction of Sláintecare (15), Ireland’s 10-year healthcare reform plan, aims to enhance access to home support services by improving integration between primary care and home care providers. The *Strategic Workforce Advisory Group* (20) has addressed workforce challenges in home care in its 2022 report, highlighting increasing demand and the need for strategic workforce planning. The proposed Statutory Home Support Scheme is expected to create a more structured and equitable framework for service provision (2). Publicly funded home support services are currently provided free of charge to recipients, unlike long-term residential or nursing home care, which requires a financial contribution from residents (3).

The introduction of the statutory scheme is anticipated to increase demand for home support services by addressing unmet needs and encouraging more people to opt for home-based care over residential alternatives. The Health Information and Quality Authority (HIQA) has released *Draft National Standards for Home Support Services* (21), which aim to improve service quality and are under public consultation. However, implementation of these changes has been delayed due to several factors, including funding challenges, workforce shortages, and the complexity of integrating existing home support services into a unified framework (22). Additionally, delays in developing the necessary regulatory and governance structures have slowed progress, as policymakers work to balance cost, accessibility, and quality of care.

#### Rising demand

Demographic shifts, particularly population aging, are placing increasing economic pressure on Ireland’s healthcare system. By 2050, the number of people aged 65 and over in Ireland is expected to nearly double, reaching 1.6 million, with those over 80 increasing from 170,000 in 2020 to 549,000 (1). This population aging trend has significant implications for workforce retention and recruitment, healthcare funding, and service delivery models. As in many developed countries, there is a growing preference for “aging in place,” with 82% of older adults in Ireland expressing a desire to receive care at home rather than in residential facilities (23). This shift necessitates significant investment in home support services to meet growing demand and ensure workforce sustainability (2). However, there is limited evidence on the types and scale of costs involved, which this paper seeks to elaborate on.

Home support services in Ireland are delivered through a mix of public and private provision, with the HSE funding a significant proportion of statutory home support directly and through HSE-approved private providers (4). As previously mentioned, in Ireland, 60–70% home support is provided through privately arranged home support services and unpaid care work (5). These informal and private care arrangements represent an important part of Ireland’s overall home support system, but they also pose challenges, particularly in terms of consistency, quality of care, and the financial sustainability of such care models (18, 19). Financial challenges persist across the system, including funding limitations, workforce shortages, and service fragmentation (20). Government policies influence the sector’s growth, with delays in implementing a Statutory Home Support Scheme affecting service stability (22).

The Irish government, through the HSE, plays a central role in funding home support services. In 2023, the government allocated €723 million to home care services, a significant increase from €487 million in 2018, reflecting the rising demand (4). However, despite these investments, challenges persist in service accessibility, waiting lists (5,863 people in 2023), and regional disparities (22). In 2023, 55,652 older people were in receipt of home support (excluding provision from Intensive Home Care Packages) this was 1% below 2022 activity but in line with 2023 targets (4). Table 1 shows figures for previous years.

**Table 1:** Home support hours provided in Ireland 2018–2023.

Home support^1^	2018	2019	2020	2021	2022	2023
People in receipt	53,000	51,345	52,881	55,043	55,675	55,652
Hours delivered	17.13 m	17.48 m	17.5 m	20.46 m	23.6 m	22.1 m

HSE Annual Report and Financial Statements 2018–2023 (4).

1 The figures exclude Intensive Home Care Packages.

Financially, the rising demand for home support services presents a challenge for long-term sustainability of the healthcare system. Although over 55,000 people receive HSE-funded home support, with over 22 million hours of care delivered, reports indicate that many eligible individuals remain on waiting lists due to funding constraints and workforce shortages (16, 19). Comprehensive data detailing the national net increase in HSWs since 2021 remains scarce and there are concerning rural disparities (24). A significant portion of the existing workforce is approaching retirement age, with 42% of care workers aged 60 and over in 2022, potentially exacerbating workforce shortages in the near future (25).

#### Estimation of additional HSWs required

To estimate the number of additional HSWs needed in Ireland, several key assumptions must be made, summarized in Table 2 (detail in Supplementary Appendix 1). First, it is assumed that 10% of the population aged 65 and over will require home support, with each person needing an average of 10 h of support per week (25). Another assumption is that a full-time equivalent (FTE) HSW works 40 h per week, or 2,080 h per year. Based on Ireland’s projected 65 + population of 1.25 million by 2040 (representing 22% of the total population) (1), and an estimation that 10% (125,400 people) will need home support, approximately 65.2 million care hours will be required annually by 2040. Accordingly, by mid-2025, Ireland will require an additional 7,069 HSWs to meet the rising demand. By 2040, the total shortfall will increase to 16,348 HSWs. This figure represents the cumulative number of additional HSWs needed between 2025 and 2040 to accommodate the growing demand for home support services. These estimates assume current service levels and workforce structures remain unchanged.

**Table 2:** Summary estimation of additional HSWs in Ireland.

Parameter	Value/assumption
Population aged 65 + by 2040	1.25 million (22% of total population)
Proportion of 65 + population requiring home support	10%
Number of individuals needing support (2040)	125,400
Average support hours per person per week	10 h
Total support hours required annually (2040)	65.2 million hours
HSW full-time equivalent (FTE) workload	40 h/week or 2,080 h/year
Additional HSWs needed by 2025	7,069
Total additional HSWs needed by 2040	16,348

ESRI, HRB (2, 5, 26).

As demand for home support services continues to rise in Ireland and other countries, the key economic challenge remains balancing funding allocations, ensuring fair wages and conditions for HSWs, while addressing the structural barriers that limit service availability such as lack of centralized routine data and digital reporting systems. An informed economic approach to funding models and policy reform will be crucial in shaping the future sustainability of home support in different countries.

#### Cost structure of home support services

Understanding the financial composition of home support services is essential for developing realistic and sustainable policy recommendations. As of 2023, the Irish government’s expenditure on home support services was approximately €723 million, with a modest increase to €725.8 million in Budget 2024. While precise cost breakdowns for Ireland’s home support system are not publicly disaggregated, available evidence suggests that the majority of expenditure is driven by labor costs. International and domestic studies consistently report that wages account for approximately 65–75% of total costs in home care services, given the labor-intensive nature of the sector (26, 27). Based on these benchmarks, an indicative cost breakdown has been constructed (see Table 3), assuming a total annual cost of €725 million. This model allocates a low 70% estimate of expenditure to HSW wages and salaries, with the remainder distributed across administration, training, travel, and miscellaneous operational and contingency costs (e.g., legal or service change costs). While these figures are estimates, they provide a useful indicative framework for evaluating policy options in the absence of detailed national expenditure data.

**Table 3:** Estimated breakdown of home support service costs in Ireland (2024, €725 Million).

Cost component	Estimated annual cost (€)	% of total cost
HSW wages and salaries	€507.5 million	70%
Administration and overheads	€101.5 million	14%
Training and professional development	€36.25 million	5%
Travel, equipment, and supplies	€36.25 million	5%
Other operational costs	€21.75 million	3%
Contingency/Miscellaneous	€21.75 million	3%
Total estimated cost	€725 million	100%

HRB unit costs of non-acute care in Ireland 2016–2019 (27), ESRI Home support services in Ireland (2022) (26).

### Home support costs compared to institutional care

Ireland’s total health budget for 2025 is €25.8 billion (28). The ESRI projects that expenditure on public and private home support services will increase substantially by 2035, with costs ranging from €1.2 billion to €3.0 billion, reflecting a 4.4 to 10.4% average annual increase (25). ESRI research has also highlighted that the economic cost of providing home care is significantly lower than institutional care (18). Comparative costs of different care models are explored below. The potential for cost savings, along with the unknown savings to the state from increased employment earnings, emphasize the economic justification for investing more in home-based care models. However, more comprehensive economic modelling, effective home support workforce planning, sector data, and sustained investment are essential to meeting population health needs and demand for community-based services.

Table 4 presents cost estimates for home support, care home support, hospital stays, and hospital-at-home services in Ireland based on data from key national sources as follows: HSE reports on home support funding and delivery (4), ESRI reports which provide insights into healthcare trends and financial projections (18, 19), Citizens Information Ireland (29) which offers detailed cost and funding information for home support and long-term care, and HIQA (21) which provides average residential care costs. Government publications, such as DoH (14, 21), further inform the estimated costs of these care models in the Irish context.

**Table 4:** Estimated home support costs compared to institutional care.

Service type	Estimated weekly cost	Cost Breakdown
Home support	€350–€700	Based on hourly rates of €15–€30 (typically 7 h of care per day). Costs vary depending on hours and type of care provided (e.g., personal care, cleaning not accommodation costs).
Care home support	€1,000–€2,500	Average weekly costs for residential care in private nursing homes. Includes room, board, and basic care services (higher rates for more intensive care (36).
Hospital stay (residential)	€2,500–€5,000	Average costs for a standard hospital bed, including accommodation and basic care (not including specialized medical treatments or surgical procedures).
Hospital at home	€1,500– €3,500	Costs for providing hospital-level care at home, including medical supervision, nursing support, and equipment. Can be more cost-effective than a hospital stay but requires significant coordination and specialist care.

Home Support: This includes personal care, cleaning, and support for daily activities, nutrition and medicines assistance but does not generally include medical treatment or interventions. The cost depends on the level and number of care hours required (typically ranging from 4 to 8 h per day, and with some people receiving 12 + including overnight support packages). Care Home Support: A higher cost option due to 24/7 residential care, which includes personal care, accommodation, and other support services that may not be available with home support. Hospital Stay: A traditional hospital stay can be the most expensive due to the high level of medical care and institutional overhead. Cost vary depending on the hospital type (public or private) and whether specialized services are required. Hospital at Home: This service provides an alternative to traditional hospital stays for patients who require medical monitoring and care but do not need the full facilities of a hospital. It can include nursing care, physiotherapy, and basic medical equipment provided in the home setting. The costs are typically lower than a hospital stay but depend on the complexity of care.

### Economic considerations of expanding the home support workforce in Ireland

#### Population needs and the current workforce

Addressing the growing demand for home support services in Ireland requires targeted policy interventions to expand and stabilize the workforce, meeting the needs of an aging population while continuing support for children and people with disabilities (30). However, the sector faces persistent challenges, including low wages, poor working conditions, limited training opportunities, and high staff turnover. A key issue is the lack of comprehensive data on population needs, particularly regarding levels of acuity, dependency, and the specific care requirements of individuals. Understanding these complexities is essential for informing workforce planning and ensuring a sustainable workforce. This, in turn, will enable the delivery of high-quality, home-based support in line with the goals of Sláintecare (15).

The home support workforce in Ireland is predominantly female, with approximately 85% of workers identifying as women (4). A significant proportion of the workforce is made up of employees from outside Ireland, with nearly 30% of home support workers coming from abroad (25). The sector is characterized by a high turnover rate, with estimates suggesting that up to 30% of workers leave within the first year (31). The workforce is largely part-time, with many workers employed on a casual basis, leading to financial instability and job insecurity (20). These factors raise questions about the cost-effectiveness of different retention and recruitment strategies, which requires further research.

#### Wages and working conditions

In 2024, the average weekly earnings in Ireland were approximately €955.49, equating to an annual salary of around €49,700 (32). The national minimum wage is €12.70 per hour (effective from January 1, 2024). The average annual salary for HSWs varies based on their employer and specific role (Table 5). For those employed by the HSE, the minimum annual remuneration is set at €30,000, up from €27,000 (in 2023), as part of efforts to address recruitment challenges in the health service (22). Agency and voluntary HSWs receive much less than HSE employed HSWs, making HSE roles more attractive financially once individuals have gained training and experience (22). Additionally, many workers are employed part-time, with inconsistent working hours that make it difficult to achieve financial stability (22). This financial instability, combined with long and irregular hours, often working alone, contributes to a high level of job dissatisfaction and burnout (20).

**Table 5:** Comparing home support work to average salaries in Ireland 2024.

Role	Average gross salary
Shop Worker (unqualified)	€21,000– €24,000
Home support worker (agency)	€20,000–€24,000
Home support worker (hse)	€27,000 - €30,000 (min. Since 2024)
Garda officer (police)	€37,000–€55,000
Teacher	€36,000–€50,000
Nurse retail manager	€33,000–€45,000 €35,000–€50,000
Care home manager	€45,000–€65,000
General practitioner	€100,000–€150,000

HSE, Irish Congress of Trade Unions; SIPTU, Salary Explorer, Indeed.

#### Recruitment and induction

Table 6 outlines estimated recruitment and induction costs for a HSW (HSE or approved private provider). The cost of Quality and Qualifications Ireland (QQI) training modules for home support workers typically ranges from €200 to €500, depending on the course and provider.^1^ Basic modules, such as those in Healthcare Support at QQI Level 5, generally cost between €200 and €300, while more advanced courses or full certifications can be priced at €400–€500. Additional fees for materials or assessments may apply, and while some employers may cover the cost, it is often the responsibility of the employee, particularly for part-time or casual workers (22).

**Table 6:** Estimated recruitment and induction costs of a home support worker.

Cost component	Estimated cost
Recruitment costs	€500–€1,500
Initial training (QQI modules)	€400–€2,000 (2–4 modules)
Induction (mentoring and shadowing)	€500–€1,000
Ongoing professional development	€200–€500 per course
Uniform (e.g., uniforms, PPE)	€100–€200
Safety equipment (e.g., gloves, masks)	€50–€150
Background checks (e.g., Garda vetting)	€50–€100
Insurance (employer liability)	€200–€400 per employee (annual)
Total estimated cost	€2,500–€5,500

HSE, ESRI, Gov.ie, CPL Healthcare, Citizen’s Information Board.

As estimated above, to maintain current levels of home support provision, Ireland will need 16,348 additional full-time equivalent HSWs by 2040. The investment required for recruitment, training, and onboarding these workers is estimated to range from €40.92 million to €89.95 million (based on a current average cost per worker of €2,500 to €5,500, as shown in table 5). These figures reflect the costs associated with bringing new workers into the sector, not their annual salaries, supervision, management or Continuous Professional Development (CPD). Thus, retention and better use of the existing workforce are clearly important, and a survey conducted by HCCI in early 2023 found that 67% of home care workers would be interested in working additional hours if social welfare rules permitted them to do so without loss of entitlements (22).

Retention of home support workers is currently a significant challenge, with turnover rates reaching as high as 30% annually (4). The primary factors driving high turnover include low wages, poor or isolated working conditions, and limited career advancement opportunities. A survey conducted by the Irish Home Care Association (33) found that 50% of workers cited low pay as their primary reason for leaving the sector, followed by burnout (42%) and lack of career progression (35%). The emotional and physical demands of caregiving, compounded by inconsistent levels of support and training, lead to high levels of burnout, particularly among new workers (34).

#### Training and skills development

Despite the demanding and often complex nature of home support work, many HSWs in Ireland hold only the basic qualifications required by the HSE or approved private providers. The QQI Level 5 in Healthcare Support is the minimum qualification for workers in this sector. It equips them with essential skills to manage home support and care needs, covering modules such as Care of the Older Person, Care Skills, Care Support, Communications, Safety and Health at Work, and Palliative Care.

O’Neil et al. (35) highlight that while specialized training is available, its uptake is often inconsistent across the sector, largely depending on individual employers’ requirements. This results in significant gaps in skills development within the workforce. Not all roles require the full suite of specialized modules (e.g., QQI Specialist Modules in Dementia Care, Palliative Care, Intellectual Disabilities Care, Autism Awareness and Support), and training typically occurs on an as-needed basis. Another challenge is the lack of centralized data on client needs and workforce skills, which is essential for ensuring home support services are adaptable and responsive to the needs of clients, particularly those with dementia (36).

Approximately **o**ne-quarter of home support workers have received formal training beyond their basic care qualifications (25). The lack of clear pathways for further education and career progression limits professional development (7). Though some QQI-accredited courses are available, access remains restricted due to financial barriers and the challenge of balancing work with study (35). This lack of access to low cost further training opportunities hampers both the quality of care provided and the long-term career prospects for HSWs (6).

### International comparisons in provision of home support

This section of the findings describes the international evidence on best practices and economic perspectives on home support, and the evidence on effective strategies such as funding models, investment strategies, local decision-making, workforce retention, training and development. By highlighting international case studies (detail in Supplementary Appendix 2), it explores how this learning can inform workforce development and policy reform to strengthen home support services.

#### Funding models

Countries use a mix of public, private, and hybrid insurance models to fund home support services and aged care. In Nordic countries, care is primarily tax-funded through universal welfare systems, ensuring broad access to home support (10). Germany and Japan operate long-term care insurance (LTCI) models, where mandatory payroll contributions fund care services, reducing reliance on general taxation (29, 30, 36) The Netherlands combines tax-based funding with social insurance, offering extensive home support benefits (37). In the UK and Ireland, aged care is funded through a mix of government subsidies and private out-of-pocket payments, often means-tested, leading to accessibility challenges (31). Many countries are shifting toward co-payment models to balance public spending and affordability while ensuring sustainability in aging societies (38).

Investment in home support plays a crucial role in workforce retention and cost-effectiveness of healthcare systems globally. A systematic review by Genet et al. (39) identifies key factors in effective home care systems, including workforce training, funding mechanisms, and integration with healthcare services. The European Commission (40) highlights career progression, fair wages, and training as essential for workforce stability. The OECD (41) reports that countries with strong home care investment experience lower turnover rates and reduced healthcare costs. In Japan, home support services are primarily funded through the Long-Term Care Insurance (LTCI) system, which provides universal coverage for adults aged 65 and older, with costs shared between government subsidies, insurance premiums, and user co-payments (42). In Canada, the CIHI (43) shows the benefits of integrated care models and targeted funding in enhancing service delivery.

#### Local needs and workforce deficits

Research in Germany shows unexplained variation in institutional (nursing home) versus home care may stem from cultural differences, individual health characteristics, diverse working arrangements, family structures (e.g., availability of informal carers), or evolving population dynamics within counties (44). While research from Australia using discreate choice experiments shows the social value of in-person home-based program for people with dementia and their carers (45). These insights suggest Ireland could improve workforce sustainability through structured local level planning, greater investment, greater engagement and partnerships with informal carers, together with professional development policies tailored to local needs.

Labor market research highlights general challenges in the home support sector, including low pay, job insecurity, and limited career progression. The International Labor Organization (ILO) (46) emphasizes the undervaluation of care work and the need for better wages and conditions to attract and retain workers. Eurofound (47) identifies workforce shortages and high turnover as key issues across Europe. King and Pickard (48) explore the impact of market-driven care systems on job stability, noting that blurred professional roles contribute to inconsistent working conditions. Addressing these challenges in Ireland requires improved job security, career pathways, and formal recognition of care work.

Recent health system reforms across Central and Eastern Europe further highlight the rapidly evolving policy landscape, as countries strive to expand community-based care and professionalize home support. For example, Poland has broadened access to home care by decentralizing services and increasing the responsibilities of local authorities (49). Hungary’s Home Care Programme offers financial assistance to families and enhances training for care workers (50). Romania’s National Strategy on Long-Term Care (2023–2030) emphasizes service quality, workforce development, and improving coordination between public and private sectors (51). Bulgaria’s Integrated Home Care Programme provides support for family carers and invests in training (52). While Croatia’s Long-Term Care Strategy (2021–2030) prioritizes home-based care through increased funding and workforce development (53).

#### Funding for training and continuous professional development (CPD)

International comparisons reveal varied approaches to funding workforce development and career structures. Sweden’s publicly funded home care system ensures professionalized career pathways, CPD, and higher wages, leading to better workforce retention (54). The Netherlands prioritizes home-based aging and workforce development but relies on a mix of public and private funding, allocating a smaller share of Gross Domestic Product (GDP) to home care than Sweden (37). In contrast, Ireland’s home support sector remains fragmented, with inconsistent training, career progression and reliance on precarious employment (7, 15, 17).

Evidence from international studies in the research literature (Table 7) highlights the importance of workforce training and development in improving capacity and care outcomes in the sector. For instance, McGilton et al. (55) demonstrated that communication training for care providers in long-term care homes improved both resident quality of life and provider well-being. Similarly, Velazquez et al. (56) explored the Health Career Access Programme (HCAP) in Canada, which offers a pathway for individuals to become Health Care Support Workers while studying, though concerns about sustainability persist. In the USA, Fong et al. (57) and Luz and Hanson (58) found that workforce training programs enhanced care quality and reduced adverse events in long-term care settings. However, studies like Rooijackers et al. (59) and Ayalon and Shinan-Altman (60) questioned the cost-effectiveness and retention outcomes of certain training interventions.

**Table 7:** Economic evidence from international case studies on home support workers.

Case study focus (country)	Evidence	Reference
Costs and benefits of workforce communication training in long-term care (Canada)	The study evaluated a communication intervention to improve communication between care providers and residents with dementia in long-term care homes. The intervention led to increased resident quality of life (QoL), improved care providers’ mood, and reduced burden. The intervention included individualized communication plans, dementia care workshops, and a support system for providers.	McGilton et al. (55)
Cost–benefit of large-scale policy directives for workforce development interventions (Canada)	The Health Career Access Program (HCAP) in British Columbia offers an innovative strategy for building a sustainable healthcare workforce. This program enables individuals to become Health Care Support Workers (HCSWs) while studying to become healthcare assistants, with all costs covered. Early feedback has been positive, and a logic model is being developed to assess long-term impact. However, the programs sustainability is uncertain due to dependence on government funding.	Velazquez et al. (2022) (56)
Cost-benefits and care quality of employment and training (USA)	Quantitative study in the USA assessing the impact of workforce training on value-based payment measures in a Medicaid-managed long-term care population in New York. Using a quasi-experimental design with data from 19,212 assessment pairs, results showed statistically significant improvements in flu vaccination rates, pain management, and shortness of breath control for different client groups. Findings suggest that workforce training could enhance care quality for high-need long-term care recipients, though its effectiveness varies by service needs. The study recommends integrating direct care workforce contributions into value-based healthcare models.	Fong et al. (57)
Cost-benefits of employment and training and reduction of costly adverse events (USA)	The “Building Training…Building Quality” (BTBQ) PCA training program aims to improve the skills, status, and job satisfaction of personal care aides (PCAs). The training program was associated with increased confidence, higher employability, and better job satisfaction. A subsequent study on the same PCA training program (BTBQ) found that clients of trained PCAs reported better health outcomes, including fewer falls and emergency department visits. This study supports the idea that better-trained PCAs lead to reduced costly adverse events and improved patient satisfaction.	Luz et al. (58) and Luz Hanson (66)
Cost-benefits of employment and training, content and methods of training (USA)	Mixed-methods study in the USA examining how the structure and culture of long-term support service (LTSS) organizations impact the effectiveness of direct care worker (DCW) training. The study used data from 328 licensed LTSS organizations in Pennsylvania. Findings suggest that training policies should focus on both the methods and content of training, addressing organizational size, evaluation practices, and DCW integration. Effective training also requires support for organizations and supervisors to enhance the learning and working environments for DCWs.	Kemeny and Mabry (61)
Cost-benefits of employment and training on career progression and occupational mobility (USA)	The study explored career transitions among individuals in entry-level healthcare occupations. Results showed limited evidence of career progression, with only a few individuals moving into higher-skilled roles. About 28% of the sample transitioned to other occupations, such as office/administrative or personal care/services. The study suggests the need for clearer career advancement pathways.	Snyder et al. (62)
Employment and training funding and impact on retention (Israel)	A 3-year evaluation of an Israeli training program for paid elder care workers. Qualitative and quantitative analysis showed strong funding and committed staff, but only 31 of 130 participants remained in the sector post-training. Key challenges included a mismatch between program vision and real-life demands, lack of role clarity, and insufficient integration of emotional/psychological care. Findings highlight the need for government-supported, top-down workforce development strategies.	Ayalon and Shinan-Altman (60)
Efficiency of organizations and care manager’s role and behaviors (Sweden)	Qualitative case study in Sweden examining the role of care managers in improving efficiency within home care organizations. Through thematic analysis of 11 interviews with care managers and 23 recruitment profiles, the study explores how managers navigate efficiency demands. Findings indicate that while care managers are aware of efficiency pressures, they do not perceive political decisions as highly restrictive. Instead, they view their role as guiding employees toward smarter resource use. This reflects a shift in home care management, emphasizing leadership behaviors that promote organizational adaptability and efficiency.	Julin (63)
Organizational capacity to implement and fulfil training agendas (England)	Part 1: Examines the career development and progression of Healthcare Support Workers (HCSWs) in the NHS, highlighting pathways to pre-registration nurse training, especially via the nursing associate role. It discusses the organizational balance needed to meet the diverse training and development aims for HCSWs. Part 2: Explores the role of functional skills in career progression for HCSWs, noting challenges such as lack of organizational funding for training, and the impact of COVID-19 on training opportunities.	Kessler et al. (67) and Kessler et al. (68)
Service market, business models, profit margins and QoL outcomes (China)	Investigates the home-based care service market in Sichuan Province, China, focusing on the conflict between supply and demand in the market and challenges arising from market-oriented operations. The study involves interviews with 350 older adult individuals to propose countermeasures and recommendations to improve resource allocation, diversify service providers, and enhance the quality of life for the older adult.	Lu (64)
Economic evaluation of reablement training and client outcomes (Holland)	The study evaluated the cost-effectiveness and cost-utility of a reablement training program for homecare staff targeting sedentary behavior in older adults. Results showed no significant differences between the intervention and usual care groups in terms of sedentary behavior, quality-adjusted life years (QALYs), or societal costs, except for lower costs for domestic help in the intervention group. The program was not cost-effective.	Rooijackers et al. (59)
Funding and resourcing, fair pay and career development (England)	Mixed-method research in England examining the social care pay gap. Findings show that social care workers earn up to 39% less than comparable public-sector roles, leading to high turnover (34% annually). Despite social care contributing £46bn to the economy and creating 1.65 m jobs annually, low pay remains a core issue. The report calls for immediate wage increases, a workforce review for fair pay benchmarks, and long-term career development strategies to stabilize the sector.	Community Integrated Care (69)
Payment models, scope of care (USA)	Qualitative study in the USA examining home health aides’ perceptions of quality care and the impact of payment models. Based on four focus groups (*n* = 27) of unionized, agency-based home health aides in New York City, the study found that current payment models focus on physical tasks, overlooking relational care that supports cognitive, emotional, and social well-being. Workers emphasized that quality care depends on strong aide-client relationships, yet they lacked supervisory support and inclusion in care teams. The study highlights the need to align payment models with the full scope of care provided, ensuring adequate support, supervision, and worker engagement in policy decisions.	Franzosa et al. (65)

Other studies, including Kemeny and Mabry (61) and Snyder et al. (62), highlight the significance of clear career progression paths and the need for effective training methods in improving job satisfaction and career mobility. Further, Julin (63) and Lu (64) reflected on the role of organizational efficiency and market dynamics in enhancing care quality. Meanwhile, Kessler et al. (53, 54) focused on the English National Health System support for support workers and the challenges posed by limited funding and the COVID-19 pandemic. Finally, Franzosa et al. (65) highlighted the misalignment between payment models and the full scope of care, suggesting a need for policy alignment that better supports home health aides’ work in delivering comprehensive care. These studies collectively emphasize the complex relationship between workforce training, organizational capacity, and client outcomes including quality of life (QoL).

## Discussion

### Areas for future research

While this study provides a broad overview of the economic dynamics shaping home support services in Ireland, several key areas warrant further investigation. First, additional empirical research is needed to validate the economic estimates presented, particularly through longitudinal data and real-world costing studies. These efforts would help to refine our understanding of the actual costs associated with service delivery, workforce inputs, and health outcomes. Second, there is a pressing need to examine the long-term sustainability of Ireland’s current funding mechanisms for home care. Future research should explore various financing models, including public, private, and hybrid approaches (end user or citizen contribution to costs), to assess their optimal viability in the context of an aging population (70, 71). There is also a need to examine the value of such services from the perspective of those people who use them, to determine which aspects of services have highest impact on quality of life or other outcomes, for example. Finally, cross-country comparative studies are essential to understanding how different health and social care systems address the challenges of home support. Further research and insights from international best practices could inform more effective policy development in Ireland, particularly in relation to workforce planning, integration with informal care, and strategic investment in community-based services.

## Policy recommendations

### Expanding and strengthening the home support workforce

Overall, the evidence suggests that to ensure a skilled and sustainable home support workforce, policy interventions can focus on improving wages, career pathways, and government investment.

*Recommendation 1) Improving Wages and Working Conditions:* Low wages and precarious employment contribute to workforce shortages and high turnover. Policies should establish a sector-wide minimum pay standard aligned with a living wage, comparable with jobs that require similar levels of training and responsibility, as seen in countries with stronger home care systems (46, 47). Enhancing employment contracts by offering minimum guaranteed hours and benefits such as paid leave and pension contributions would improve job security and retention (41).

*Recommendation 2) Establishing Career Pathways and Professional Development:* A structured career pathway is essential for the attractiveness of the HSW roles, cost savings associated with retention, and professionalization. The evidence and international best practice suggests Ireland should introduce a tiered certification framework, where progression to advanced or more specialized roles—such as senior support workers or care coordinators for specific client groups—is linked to further qualifications (40). Expanding government-funded training programs, including subsidized QQI-accredited courses, and accessible CPD and career development tools online, would reduce financial barriers to upskilling (39).

*Recommendation 3) Increased Government Investment:* Sustained public funding is necessary to ensure the quality and accessibility of home support services. Increased investment in home care could alleviate pressure on institutional healthcare, leading to long-term healthcare cost savings (43). The longterm funding strategy should prioritize service delivery, research and workforce development, ensuring financial support for training and fair remuneration (54).

For Ireland and any other country, implementing these policy recommendations requires a coordinated, responsive long-term strategy that recognizes home support as an integral component of the broader health and social care system. Addressing workforce issues in isolation will be insufficient without parallel reforms that improve service integration, recognize the role of informal carers and communities, and ensure equitable access to quality care across regions. Policymakers must also consider the fiscal implications of these recommendations, framing investment in home support not simply as a cost, but as a means to achieve greater efficiency, better outcomes for older people, and reduced demand on acute and institutional care. Embedding home support within national aging and health workforce strategies, supported by ongoing research, monitoring, and evaluation, will be essential to ensure these reforms are both economically sound and socially responsive.

## Conclusion

This research provides comprehensive insights into the economics of delivering home support services in Ireland, shaped by an aging population and growing demand for care at home. The evolving demographic and health landscape underscores the need for a more sustainable, structured approach to the funding and organization of home care services—both in Ireland and in other countries facing similar pressures. Policy action is critical, particularly in addressing the challenges confronting the home support workforce, such as low pay, job insecurity, and limited opportunities for career progression. These factors contribute to high staff turnover and constrain the sector’s capacity to meet current and future demand. International comparisons show that investment in clear career pathways, fair wages, and robust training can enhance workforce stability, reduce reliance on institutional care, and expand overall system capacity. Further research is needed to examine the assumptions and evidence underpinning financial decision-making, to support the development of sustainable and effective home support services in Ireland and other countries.

## Data Availability

The original contributions presented in the study are included in the article/Supplementary material, further inquiries can be directed to the corresponding author.

## Footnotes

^1^ https://www.qqi.ie/fees-schedule
